# Spontaneous Regression of Cranial Vault Lymphoma: A Case Report

**DOI:** 10.7759/cureus.87472

**Published:** 2025-07-07

**Authors:** Kazutaka Kishaba, Yohei Hokama, Tomomi Kuninaka, Hirofumi Miyahira, Mariko Tomita, Naoki Wada, Akira Yogi, Tadashi Hamasaki

**Affiliations:** 1 Neurosurgery, University of the Ryukyus Hospital, Ginowan, JPN; 2 Diagnostic Pathology, University of the Ryukyus Hospital, Ginowan, JPN; 3 Pathology and Oncology, Graduate School of Medicine, University of the Ryukyus, Ginowan, JPN; 4 Radiology, Graduate School of Medicine, University of the Ryukyus, Ginowan, JPN; 5 Neurosurgery, Graduate School of Medicine, University of the Ryukyus, Ginowan, JPN

**Keywords:** biopsy, cranial vault, lymphoma, spontaneous regression, watch and wait

## Abstract

Primary lymphoma of the cranial vault is extremely rare and often mimics other cranial bone tumors on imaging. We report a 75-year-old female with a scalp mass and occasional headache. MRI revealed a lesion centered in the cranial vault, extending both intra- and extracranially. The initial diagnosis was intraosseous meningioma, but the lesion rapidly decreased in size over a few weeks without any treatment. Biopsy revealed histology consistent with non-Hodgkin lymphoma. Further classification suggested marginal zone B-cell lymphoma, a rare entity arising in the cranial vault. We managed the patient with imaging follow-up and found no recurrence for six months. The present case supports the notion that indolent lymphomas arising in the cranial bone share regression potential seen in other tissues. Accurate diagnosis requires histopathological confirmation even when lesions regress spontaneously. A watch-and-wait approach may be reasonable for selected cases, with careful monitoring for progression or transformation.

## Introduction

The diagnosis of malignant lymphoma arising in the cranial vault presents a significant clinical challenge due to its extreme rarity [1,2]. The majority of primary cranial bone tumors are known to occur at the skull base [3], making lesions in the cranial vault an unusual presentation. Among primary bone lymphomas, which themselves constitute only about 1% of all malignant lymphoma cases, those originating from cranial bones account for approximately 5.5% [4]. Owing to this rarity and their atypical anatomical location, cranial vault lymphomas often go unrecognized in early stages and may be frequently misdiagnosed. Clinically and radiologically, cranial vault lymphomas commonly mimic more prevalent skull lesions such as intraosseous meningiomas, due to overlapping features such as subcutaneous mass formation and enhancement patterns on imaging [1,5-7]. However, one reported distinguishing feature is the relatively rapid growth of the lesion over several months, which may aid in their differentiation from other indolent skull tumors [1,2].

Spontaneous regression or remission, defined as the partial or complete disappearance of a lesion without specific medical intervention, is a recognized but uncommon phenomenon seen in indolent histological subtypes of non-Hodgkin lymphoma. It has been reported to occur in approximately 10-20% of cases in selected series [8]. For instance, marginal zone B-cell lymphoma (BCL) of mucosa-associated lymphoid tissue (MALT) in the conjunctiva has demonstrated a high rate of spontaneous regression [9], and there are also rare reports of spontaneous regression in lymphomas coexisting with solid tumors, such as breast carcinoma [10].

Despite these observations in other anatomical sites, spontaneous regression has not been previously recognized as a characteristic of lymphomas arising in the cranial vault. We present here a rare case of primary cranial vault lymphoma in an elderly woman, which underwent spontaneous regression without medical treatment. To our knowledge, this is the first report documenting such behavior in this anatomical location.

## Case presentation

**Figure 1:**
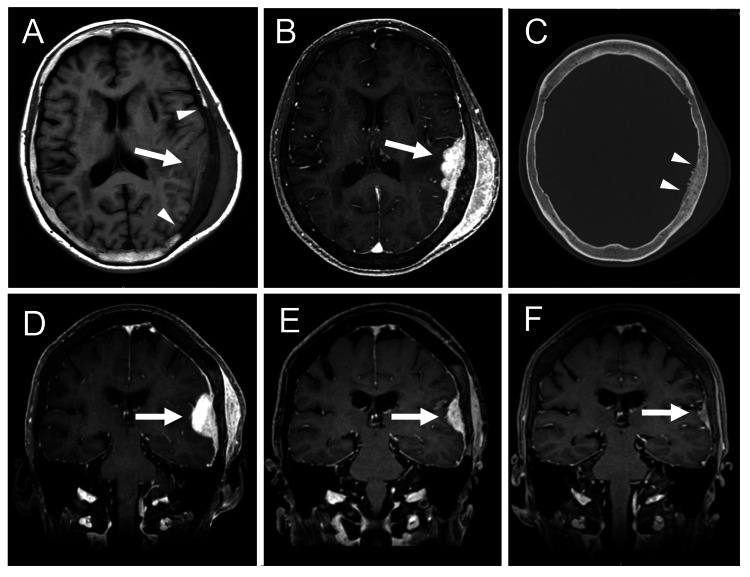
MR imaging and CT scan in the clinical course. (A) An axial slice of T1-weighted MR image at the initial visit. An arrow indicates an isointense mass lesion attached to the cranial vault. Arrowheads indicate clear boundaries of the lesion in the bone marrow. (B) An axial slice of gadolinium-enhanced MR image at the initial visit. An arrow indicates the lesion extending intracranially and extracranially and well-enhanced with gadolinium. (C) A CT scan of the cranial bone at the initial visit. Arrowheads indicate hyperostosis in the inner surface of the bone involved in the lesion. (D-F) Coronal sections of MR images obtained at the initial visit (D), a few weeks later (E), and at the latest follow-up (F). Arrows indicate the lesion in the cranial vault that decreases in size spontaneously.

**Figure 2:**
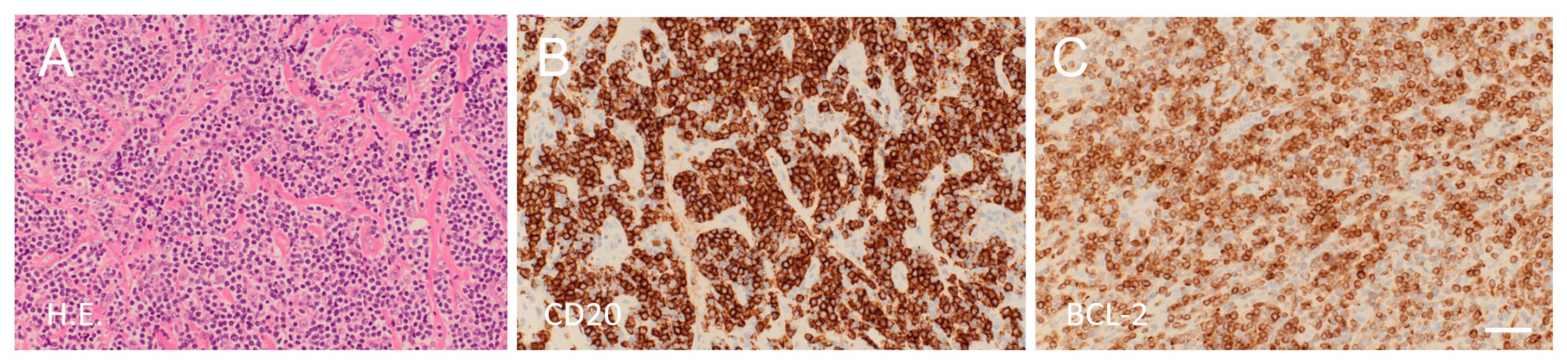
Histopathology of the biopsy specimen. (A) Hematoxylin and eosin staining. Small to medium-sized lymphoid cells are diffusely infiltrated. These cells exhibit centrocyte-like cell or monocytoid cell morphology. (B) CD20 Immunostaining. The tumor cells are immunopositive for CD20. (C) BCL-2 Immunostaining. The tumor cells are immunopositive for BCL-2. Scale bar, 100 mm for A-C. Original magnification, ×200 for A-C. BLC, B-cell lymphoma; CD, cluster of differentiation

This 75-year-old female had a past history of cured breast cancer, temporomandibular joint disorder, and varicose veins. She had been taking no medication for several years. She was referred to our hospital after MR imaging, performed to evaluate occasional headaches over the past few weeks, showed a lesion in the cranial vault. Physical examination revealed a soft, painless subcutaneous mass palpable in the left vertex of the head. She was alert and well-oriented. Neurological examination was normal except for occasional light headaches. Head MRI demonstrated a mass lesion in the cranial bone of the left convexity extending both intracranially and extracranially, which was well- and uniformly enhanced with a contrast medium (Figures 1A-1B). Head CT showed hyperostosis in the inner surface of the cranial bone involved in the lesion (Figure 1C). General blood test and tumor markers, including CA15-3, CEA, AFP, CA-125, CYFRA, CA19-9, SCC, and PIVKA-II, were normal. A whole-body CT scan showed no evidence of recurrent breast cancer or any other malignancy, such as lung or thyroid cancer. Fluorodeoxyglucose positron emission tomography showed no hypermetabolic mass in the whole body other than the cranial bone lesion. We preoperatively diagnosed intraosseous meningioma and planned surgical removal. Unexpectedly, repeated MRI performed a few weeks later demonstrated spontaneous regression (Figures 1D-1F). We performed a biopsy of the subcutaneous portion of the lesion under local anesthesia, as a definitive diagnosis was necessary. Hematoxylin and eosin staining of the specimen revealed a dense, nodular to diffuse infiltrate of small to medium-sized lymphoid cells in areas where the lesion extends into the subcutaneous tissue (Figure 2A). These cells exhibited inconspicuous nucleoli and centrocyte-like cell or monocytoid cell morphology. Immunostaining showed that the tumor cells were positive for CD20 (Figure 2B) and BCL-2 (Figure 2C), and that a minor component of the tumor cells was undeniably BCL-6 positive. Immunostaining also showed that the tumor cells were negative for Cyclin D1 and TdT. To evaluate the presence of clonal lymphoproliferation, a polymerase chain reaction (PCR)-based analysis of immunoglobulin gene rearrangements was performed using the GeneScan method [11]. Consequently, monoclonal growth patterns were observed in the rearrangement of immunoglobulin kappa (IGK) genes, specifically Vκ-Jκ and Vκ/intron recombination signal sequence (RSS)-Kappa-deleting element (Kde) recombinations, against a background of polyclonal proliferation. These findings were consistent with non-Hodgkin lymphoma, and the histological classification was suggestive of marginal zone BCL, although plasmacytic differentiation was less obvious, and a minor component of the tumor cells was undeniably BCL-6 positive.

The postoperative course was uneventful. We managed the patient with a watch-and-wait strategy, based on previous reports of marginal zone BCL in other tissues [9,12-14]. With regular imaging follow-up to monitor for tumor recurrence, the patient remained asymptomatic for six months.

This study was approved by the Ethics Committee of the University of the Ryukyus for Medical and Health Research Involving Human Subjects (approved #: 24-2410-00-00-00). A written informed consent for publication was obtained from the patient.

## Discussion

We present a case that underwent spontaneous regression of cranial vault lymphoma in the absence of medical intervention. Spontaneous regression or remission has been observed in lymphomas arising in other tissues [8-10]; however, such behavior has not been documented in lymphomas originating in the cranial vault. Previous reports suggested that spontaneous regression was seen particularly under specific clinical conditions, including discontinuation of immunosuppressive agents [15,16], eradication of *Helicobacter pylori* infection in the stomach [17], and use of antibiotics [18]. The mechanisms underlying spontaneous regression in our case are unknown because she had no medication and none of the conditions described above. Apoptosis of tumor cells, immune reactions in the host, or particular conditions of the tumor microenvironment may play a role [19].

Our initial diagnosis was intraosseous meningioma. Although the histology of tumors arising in the cranial vault varies, meningiomas, metastatic carcinomas, and histiocytosis are reportedly more common [20,21]. The initial symptoms are expected to be similar due to the shared site of tumor origin. Previous reviews on cranial vault lymphoma reported that the initial symptom is a subcutaneous scalp mass, headache, focal neurological deficit, and seizure [1,2,22], which are similar to those of intraosseous meningioma [7]. Imaging features in MRI, including iso- or hyper-intensity in T2-weighted image and enhancement by contrast medium, also mimic intraosseous meningioma. Skull X-rays and CT scans may be useful for differential diagnosis, showing osteolysis in 74-76% of cranial vault lymphoma [1,2] in contrast to hyperostosis in about 59% of meningioma [23]. A previous review suggested that unique features that support the diagnosis of lymphoma were a history of rapid growing subcutaneous mass in several months [1,2,6], poor vascularization in angiogram [6], and limited bone destruction in CT scan [1,2]. The present case demonstrated spontaneous regression, which is also observed in other cranial vault tumors such as Langerhans cell histiocytosis [24], which is also a characteristic supporting the diagnosis of lymphoma.

Previous reports described that cranial vault lymphoma was effectively managed by chemotherapy, including R-CHOP (rituximab, cyclophosphamide, doxorubicin hydrochloride, vincristine, prednisolone) and radiation therapy [1,2,5,6,22]. According to the review by Nitta et al. [1], survival time after radiotherapy, chemotherapy, or a combination of them for cranial vault lymphoma, most of whom had diffuse large B-cell histology, was from two weeks to 13 years. Is it appropriate to manage our case with biopsy and imaging follow-up? Matsuo and Tanaka reported long-term observation without treatment in extra-nodal marginal zone BCL of MALT arising in the conjunctiva [9]. The authors managed 24 of 31 patients by observation and found only five of these 24 patients relapsed in 0.5 to six years [9]. A watch-and-wait approach reportedly has no adverse impact on overall survival when marginal zone BCL arises in the spleen [12]. A watch-and-wait approach is also an option for asymptomatic nodal marginal zone BCL [13]. Thus, imaging follow-up is a reasonable approach in our case, as long as we acknowledge the possibility of histological transformation to diffuse large BCL, which occurs at an annual incidence of approximately 1% [25]. We suggest that biopsy should be considered even for asymptomatic tumors that spontaneously regress prior to diagnosis, in order to determine an appropriate therapeutic approach.

## Conclusions

We report a rare case of cranial vault lymphoma that exhibited spontaneous regression without any medical intervention. This phenomenon, while described in other tissues such as the conjunctiva, has not been reported previously in lymphomas arising in the skull bone. Clinical and radiological features in the present case mimicked intraosseous meningioma, complicating initial diagnosis. Biopsy revealed marginal zone BCL, an indolent subtype known for its potential to regress spontaneously. We managed the patient with a watch-and-wait approach, and the patient remained asymptomatic and recurrence-free for six months. Given the potential for misdiagnosis, we suggest that histological confirmation should be considered even in spontaneously regressing tumors in the cranial vault. This case contributes to the limited literature on cranial vault lymphoma and suggests that spontaneous regression, although rare, may occur in indolent forms of lymphoma in this region. Imaging follow-up is essential to monitor for recurrence or histological transformation, which can alter the therapeutic strategy.
